# Adaptation of *Propionibacterium freudenreichii* to long-term survival under gradual nutritional shortage

**DOI:** 10.1186/s12864-016-3367-x

**Published:** 2016-12-08

**Authors:** Flavia Figueira Aburjaile, Marine Rohmer, Hugues Parrinello, Marie-Bernadette Maillard, Eric Beaucher, Gwénaële Henry, Aurélie Nicolas, Marie-Noëlle Madec, Anne Thierry, Sandrine Parayre, Stéphanie-Marie Deutsch, Muriel Cocaign-Bousquet, Anderson Miyoshi, Vasco Azevedo, Yves Le Loir, Hélène Falentin

**Affiliations:** 1 Department of General Biology, Federal University of Minas Gerais, Belo Horizonte, Minas Gerais Brazil; 2 INRA, UMR 1253, Science et Technologie du Lait et de l’ Œuf, 35000 Rennes, France; 3 AGROCAMPUS OUEST, UMR1253, UMR Science et Technologie du Lait et de l’Œuf, 35000 Rennes, France; 4 UMS BioCampus- MGX Montpellier GenomiX, Institut de Génomique Fonctionelle, 141, rue de la Cardonnille, 34094 Montpellier Cedex 05, France; 5 LISBP, Université de Toulouse, CNRS, INRA, INSA, 135 avenue de Rangueil, Toulouse, 31077 France

**Keywords:** *Propionibacterium freudenreichii*, Long-term survival, Stationary phase, RNA-seq, Adaptation

## Abstract

**Background:**

*Propionibacterium freudenreichii* is an Actinobacterium widely used in the dairy industry as a ripening culture for Swiss-type cheeses, for vitamin B12 production and some strains display probiotic properties. It is reportedly a hardy bacterium, able to survive the cheese-making process and digestive stresses.

**Results:**

During this study, *P. freudenreichii* CIRM-BIA 138 (alias ITG P9), which has a generation time of five hours in Yeast Extract Lactate medium at 30 °C under microaerophilic conditions, was incubated for 11 days (9 days after entry into stationary phase) in a culture medium, without any adjunct during the incubation. The carbon and free amino acids sources available in the medium, and the organic acids produced by the strain, were monitored throughout growth and survival. Although lactate (the preferred carbon source for *P. freudenreichii*) was exhausted three days after inoculation, the strain sustained a high population level of 9.3 log_10_ CFU/mL. Its physiological adaptation was investigated by RNA-seq analysis and revealed a complete disruption of metabolism at the entry into stationary phase as compared to exponential phase.

**Conclusions:**

*P. freudenreichii* adapts its metabolism during entry into stationary phase by down-regulating oxidative phosphorylation, glycolysis, and the Wood-Werkman cycle by exploiting new nitrogen (glutamate, glycine, alanine) sources, by down-regulating the transcription, translation and secretion of protein. Utilization of polyphosphates was suggested.

**Electronic supplementary material:**

The online version of this article (doi:10.1186/s12864-016-3367-x) contains supplementary material, which is available to authorized users.

## Background

*Propionibacterium freudenreichii* is an Actinobacterium widely used in the dairy industry and responsible for aroma development and opening (eyes) in Swiss-type cheeses. Some strains are also used as probiotics because they produce bifidogenic compounds [1], they are resistant to digestive stress [2] and they may be endowed with anti-inflammatory capabilities and could be used to prevent inflammatory bowel diseases [3] [4]. *P. freudenreichii* core metabolism leads to propionic acid as the main end-product [5].

The genetic bases for hardiness have already been well-documented. *P. freudenreichii* can produce ATP and NAD(P)H via fermentation utilizing an unique metabolic pathway (the Wood-Werkman cycle), leading to propionic acid. Under anaerobiosis, *P. freudenreichii* can also use electron acceptors other than O_2_ (such as humic acid or nitrate) to produce ATP during anaerobic respiration [5, 6]. Several genes involved in polyphosphate and pyrophosphate utilization are found in the genome of CIRM-BIA1, the type strain for *P. freudenreichii*, suggesting a storage of energy in these forms [7]. An inorganic pyrophosphatase-coding gene was found to be overexpressed in the cold in 6 strains of *P. freudenreichii* [8]*. P. freudenreichii* can stay alive and metabolically active for long periods even under stressful conditions like gastro-intestinal tract environment or in cold condition. When placed at stationary phase in the intestinal tract (colon) of a piglet, CIRM-BIA1 was able to ferment some atypical carbon sources present in the gut (gluconate and propanediol), enabling its survival under these harsh conditions. Both of these catabolic pathways were reconstructed *in silico* from the genomic data and were found to be fully expressed in the colon of piglets [9]. Another transcriptomic study on the adaptation of *P. freudenreichii* strains to the cold (with lactate fully available) showed that strains slowed down their cellular metabolism, displayed cold stress responses, and rerouted their carbon metabolism toward trehalose and glycogen synthesis [8]. In addition, proteomic studies on different *P. freudenreichii* strains have highlighted the role of chaperones during exposure to acids, bile salts or NaCl [10–12]. The genomic analysis of *P. freudenreichii* strains thus revealed the duplication of several chaperone genes.

In a previous study [13], we carried out the phenotypic characterization of *P. freudenreichii* over an 11-day period without the addition of nutrients, and revealed different phases of growth, membrane permeabilization and entry into dormancy and a viable but non-culturable state to ensure Long-Term Survival (LTS). How *P. freudenreichii* prepares the LTS phase remains unknown and no data are currently available on the metabolism of *P. freudenreichii* in stationary phase in the event of a gradual nutritional shortage.

The objective of the present study was therefore to identify the pathways used by *P. freudenreichii* to cope with starvation and enter the LTS phase. The strategy adopted here combined the quantification of sugars, acids and free amino acids in the supernatant of an 11-day culture without the addition of nutrients, and an RNA-seq analysis of bacterial cells sampled at three different time points during *P. freudenreichii* culture. The studies were performed on CIRM-BIA138 strain because it was the strain with the highest survival rate in stationary phase, according to a screening of 23 strains [13]. This work was designed to compare both biochemical quantifications (acids, amino-acids and sugars) and transcriptomic data between the exponential and stationary phases in order to gain an in-depth view of *P. freudenreichii* adaptation to LTS.

## Results and discussion

The objective of the study was to identify the metabolic pathways used by *P. freudenreichii* to cope with starvation and ‘prepare’ entry into the LTS phase.

### Enumeration, DO, pH

**Fig. 1:**
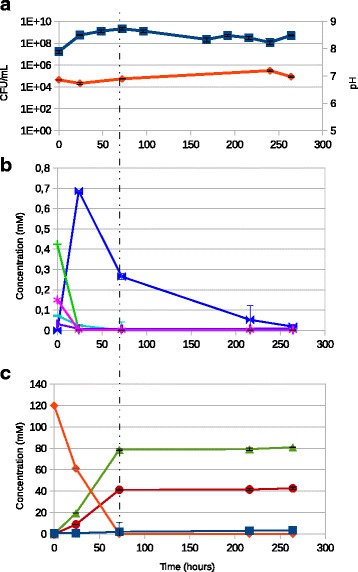
DO, CFU, pH, sugar utilization and organic acid consumption and production during the growth and long term survival over 11 days of CIRM-BIA138 in YEL medium. **a** Bacterial enumeration (CFU/mL), and pH (**b**) Quantification of sugars (glucose: pink stars, mannose: purple bold pipes, fructose: light blue dashes, lactose: green pipes) for conversion to pyruvate (blue bow tie). **c** Quantification of acids (succinate: dark blue square, lactate: orange diamond, acetate: red circle, propionate: green triangle). The vertical dotted line indicates entry into stationary phase (3d post-inoculation). Each quantification was repeated three times, standard deviations were indicated for each point

The results of bacterial enumerations and pH measurements (Fig. 1a) confirmed that CIRM-BIA 138 survived well (around 8 log_10_ CFU/mL) during 11 days, even under conditions of nutritional shortage, as it had been observed during a previous study [13]. When inoculated at 7 log_10_ CFU/mL, the *P. freudenreichii* culture entered the exponential growth phase to reach a maximum population of 9.3 log_10_ CFU/mL 3d post-inoculation, corresponding to entry into the stationary phase. The pH was very slightly affected by growth since the most acidic point was 6.7 compared to 7 at inoculation time. At entry into stationary phase, a slight decline in the population was observed until it stabilized at around 8.5 log_10_ CFU/mL as from 9d post-inoculation (Fig. 1a).

### Carbon source utilization, organic acid utilization and production

*P. freudenreichii* CIRM-BIA138 can degrade glucose, glycerol, mannose, galactose, inositol, erythritol, adonitol, esculine, lactose, xylitol, gluconate, D-fructose and L-arabinose [14]. In this study, CIRM-BIA138 was cultivated in Yeast Extract Lactate, a growth medium containing 120 mM lactate. Small quantities of several other carbon sources were also identified: 0.42 (+/- 2E-4) mM lactose, 0.15 (+/- 2E-4) mM glucose, 0.07 (+/- 3E-5) mM fructose, 0.03 (+/- 5E-5) mM mannose. Inositol and arabinose were not detected and probably below the detection limit of 0.5 mM. *P. freudenreichii* CIRM-BIA138 used lactose, glucose, mannose and fructose at the beginning of growth until they were completely exhausted at 1d post-inoculation (Fig. 1b). These degradation capacities are in line with those previously described for this strain [15]. The degradation of these sugars was concomitant with the release of pyruvate into the medium, this being one of their intermediary metabolites. From 1d post-inoculation onwards, pyruvate was consumed by the strain. Levels of other compounds such as propionate, acetate and succinate rose gradually in the medium over time during growth, to reach a plateau at 3d post-inoculation (Fig. 1C). The stoichiometry of the conversion was 3 mol of lactate into 1 mol of acetate and 2 mol of propionate, and is in line with those described previously [14]. Lactate levels decreased as early as the onset of growth because of its consumption by *P. freudenreichii*. Lactate was exhausted 3d post-inoculation and pyruvate 11d post-inoculation. Among all the carbon sources quantified during this experiment, pyruvate was the only carbon source detectable in the medium when *P. freudenreichii* entered the stationary phase.

### Overview of differentially expressed genes between exponential and stationary phases

**Fig. 2:**
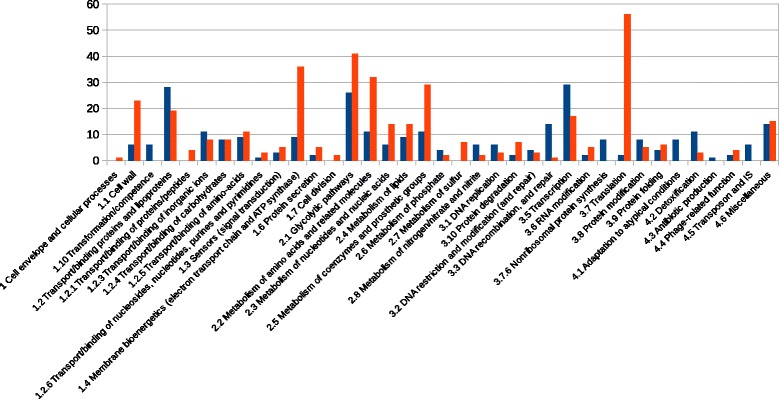
Number of induced and repressed genes in the stationary phase (3d post-inoculation) compared to the exponential phase (1d post-inoculation), classified by metabolic function. In blue and orange: induced and repressed genes, respectively. Differential comparison of groups (each containing three repetitions) were performed gene by gene using a modified *t*-test. Genes were declared as being differentially expressed (DE) with a *P* value adjusted ≤0.05

After filtration to eliminate reads of poor quality, sequencing generated 100,450,770 reads (FastQC results), which included 13,502,207 non-aligned reads and 37,018,311 reads aligned on coding sequences. The remaining reads corresponded to those mapping on two different sequences (1,147,269 ambiguous reads according an htseq-count with a union parameter), on tRNA, rRNA, or non coding RNA (small RNA, non-sense RNA, 5′ and 3 ′ UTR). Only reads mapping on coding sequences were further analysed. Overall, 912 genes were found to be differentially expressed (adjusted pvalue < 0.05) between 1d (exponential phase) and 3d post-inoculation (start of stationary phase) (458 genes induced (Additional file 1: Table S1), 454 genes repressed (Additional file 2: Table S2). Differentially expressed genes (adjusted pvalues < 0.05, pvalues available in Additional files 1: Table S1, Additional files 2: S2 and Additional files 3: S3) represented 40% of the protein-coding genes in the CIRM-BIA138 genome. The genes most affected by entry into stationary phase was those implicated in carbohydrate metabolism with 26 genes of this functional category being induced and 41 repressed. Translation was the second most affected category, with two genes induced and 56 repressed. Transport/binding was the third most affected category, with 28 genes induced and 19 repressed. Transcription was the fourth most affected category, with 29 genes induced and 17 repressed. Membrane bioenergetics was the fifth most affected category, with nine genes induced and 36 repressed. Amino acid metabolism category was also affected, with 11 genes induced and 32 repressed. Lastly, the metabolism of coenzyme and prosthetic groups was also affected, with 11 genes induced and 29 repressed (Fig. 2). Most induction and repression observed from RNAseq analysis were confirmed by RT-qPCR analysis (*p* value < 0.05, Additional file 3: Table S3). To validate RNAseq results, we performed RT-qPCR and we included the 9d post-inoculation sample. In most cases, the tendency (induction versus repression) seen at 3d post inoculation was confirmed at 9d. A massive number of differentially expressed genes (63 repressed and 177 induced) were annotated as having an “unknown function”. 39% of induced genes at stationary phase were of unknown function. The genome of CIRM-BIA 138 contains 27% of genes encoding protein of unknown function (annotated genome available at http://www.ebi.ac.uk/ena/data/view/PRJEB6433). So, results suggested that genes expressed at the entry into stationary phase are enriched in genes without function, thus reflecting our lack of knowledge on bacterial stationary phase metabolism. All induction and repression discussed below are statistically significant at adjusted pvalue < 0.05 for RNAseq and at pvalue < 0.05 for RT-qPCR.

### Slowdown of the metabolism leading to dormancy

RNA extraction and quantification throughout growth and survival showed that RNA levels gradually declined. The total/depleted RNA quantities corresponding to 2 mL of culture were 60 μg/290 ng at 1d post-inoculation (exponential growth phase) for 15 log_10_ CFU, 22 μg/225 ng at 3d post-inoculation (entry into stationary phase) 19 log_10_ CFU and 15 μg/90 ng at 11d post-inoculation 18 log_10_ CFU. The translation machinery was also reduced at 3d post-inoculation. Indeed, expression of the translation initiation factor IF-3, and the elongation factors Tu and G encoded by genes *infC*, *tuf*, *fusA,* were 2, 1.8 and 3-fold repressed, respectively. The expression of ribosomal proteins was repressed from 1.5 to 16 fold (see Additional file 2: Table S2, for repression fold and pvalue of each gene). The non coding RNA named ‘pseudoknot of the domain G(G12) of 23S ribosomal RNA’ was also repressed with a fold change of 3.7 at the entry into stationary phase (Additional file 2: Table S2). Taken together, these repressions reflected a slowdown of the cellular machinery in line with the process of dormancy recently described [13]. This slowdown can be explained by the scarcity of carbon sources when *P. freudenreichii* enters the stationary phase at 3d post-inoculation. The main glycolytic pathways producing reducing equivalents were repressed during this phase. In YEL medium, glycolysis, the pentose phosphate pathway and the Wood-Werkman cycle (fermentation) are three ways for *P. freudenreichii* to produce NADH and NADPH reducing equivalents. ATP and precursor metabolites are required for the biosynthesis of essential compounds (amino acids, purine, pyrimidine, glycerol 3 phosphate, fatty acids, N-acetyl glucosamine, vitamins). Here, under the conditions we applied, glucose was exhausted at 3d post-inoculation, and the genes involved in glycolysis were repressed. Some of them were particularly strongly down-regulated, such as *sglT*, the glucose transporter with a fold change of 4, and *pfkA*, *pfp*, *fba1* and *eno2* with fold changes of 2.2, 3.1, 1.9 and 3.4, respectively. The repression of *sglT* was confirmed by RT-qPCR results (pvalue < 0.05) with a repression fold of 6 at 3d and 88 at 9d post-inoculation (Additional file 3: Table S3).

The Wood-Werkman cycle is specific to certain propionic acid-producing bacteria. It plays a central role in propionic acid fermentation, the principal carbon metabolic pathway in dairy propionibacteria. This pathway provides major sources of reducing equivalents: NADPH required for biosynthesis reactions and NADH necessary for oxidative phosphorylation. Through this pathway, pyruvate is converted into propionate. Pyruvate is initially converted into succinate by the successive steps of the tricarboxylic cycle (TCA). At 3d post-inoculation, gene-set enrichment analysis identified the “Citrate Cycle” as one of the most down-regulated pathways (ko00020, fold change of 2.1, *p* value = 2.7e-04). All the corresponding genes (*mdh*, *fumC* and *sdh*) were repressed, with fold changes of 1.9, 2.1 and 8.6, respectively, in stationary phase compared to exponential phase (see Additional file 2: Table S2). Succinate is then converted into succinyl-CoA, methyl malonyl CoA, propanoyl CoA and propionate by specific enzymes. Transcripts corresponding to the 12S, 5S and 1.3S subunits of the well-studied methylmalonyl-CoA carboxyltransferase were repressed with fold changes of 2.3, 1.8 and 2.9, respectively. RT-qPCR results confirmed the repression of *mmda*, encoding the 12S subunit with a fold change of 2. Down-regulation of the Wood-Werkman cycle is probably involved in maintaining the redox balance. Although glycolysis and the Wood Werkman cycle were down-regulated, all genes in the pentose phosphate pathway (except the *rpiB3* gene, induced with a fold change of 2.9) were stably expressed between 1d and 3 d post-inoculation.

### Changes in oxidative phosphorylation

Several transcripts (*nuoA,B,C,D,E,F,I,J,K,L,M,N* genes) encoding the different chains of NADH-quinone oxidoreductase (responsible for the release of electrons and H+ contained in the NADH molecule) involved in aerobic respiration were down-regulated at 3d post-inoculation, with a fold change of between 3.8 and 6.3. The PFCIRM138_05930 gene encoding an iron-sulphur protein was also down-regulated, with a fold change of 6 in RNAseq analysis and 4.5 at 3d and 5.5 at 9d post- inoculation according to RT-qPCR results. Likewise, *sdhA, A3, B, B3, C1, and C2*, encoding the different subunits of succinate dehydrogenase, were down-regulated with fold changes of between 4.4 and 8.8 (for details, see Additional file 1: Table S1 and Additional file 2: S2). Repression of *sdhC1* was confirmed by RT-qPCR results with a repression with a fold of 9 (Additional file 3: Table S3). By contrast, both the *dmsC* and *dmsB* genes, encoding the anaerobic dimethyl sulphoxide reductase chains C and B, were induced with fold changes of 13.7 and 4.7, respectively. The latter protein handles the final transfer of electrons to various sulphoxide and N-oxide compounds. Anaerobiosis-inducible dimethyl sulphoxide reductases play a key role in bacterial adaptation to anaerobic conditions in bacteria and serve as terminal reductases using DMSO as a terminal electron acceptor [16]. Nitrate, sulphur or ferrous ions can act as electron acceptors. Accordingly, the gene encoding the permease protein of the nitrate ABC transporter *ssuC* was induced with a fold change of 4.4, the gene *citT2* encoding a sodium:sulphate symporter was induced with a fold change of 4.3, and the *feuC* and *feuS* genes encoding the ferrous ABC transporter were induced with fold-changes of 2.5 and 2.8. Some subunits of nitrate reductase, namely those encoded by *narH* and *narJ,* were induced by more than 22-fold. RT-qPCR results confirmed the induction of *narH* with a lesser fold of 3.5. During growth, the headspace of the tube contained air and was therefore a source of O_2_. Although the culture was grown without agitation, the medium contained traces of O_2,_ which were probably used as a terminal electron acceptor during the exponential growth phase. At 3d post-inoculation, the CO_2_ released by the bacteria likely saturated the headspace of the tube and the *P. freudenreichii* were in anoxic conditions. This might modify the redox balance due to the lower availability of reducing equivalents, and thus explain the down-regulation of oxidative phosphorylation. At the pathway level, gene-set enrichment analysis (see Methods) showed that oxidative phosphorylation was indeed the most significantly down-regulated (ko00190, fold change of 4.4 and pval = 1.2e-12) of all of the pathways down-regulated during the study. A similar down-regulation had been observed when *P. freudenreichii* was placed in the colon of piglets under anoxic conditions [9]. To assess whether the oxydative repression is due to traces of O_2_ in the headspace or due to the metabolism at the entry into stationary phase, cultures growing in bioreactors under strictly controlled conditions can be considered.

The changes thus observed suggest profound metabolic reprogramming in response to starvation. Similarly, during our study, specific catabolic pathways were induced (see below).

### Cell wall

The cell envelope of Gram positive bacteria such as *P. freudenreichii* comprises the inner cell (cytoplasmic) membrane and the cell wall of the bacterium composed of peptidoglycan and various associated compounds (proteins, polysaccharides, teichoic acids) that differ from one species to another. In *P. freudenreichii*, some strains are known to possess a surface exopolysaccharidic (EPS) layer composed of (1 → 3,1 → 2)-β-D-glucan [17].

Peptidoglycan (PG) is essential to maintaining cell shape and also providing mechanical protection against osmotic pressure. It is also involved in cell division process. PG is a three-dimensional network made up of N-acetylglucosamine (GlcNAc) and N-acetylmuramic acid (MurNAc). The carboxyl groups of MurNAc are substituted by a short peptides, interconnecting the chains together. The biosynthesis of PG includes cytoplasmic steps mediated by MurA to F, and thereafter the transfer through the membrane via the transferases MarY and MurG, and finally the binding of new material to cell wall. At entry into stationary phase, the PG biosynthesis pathway is significantly down-regulated. The genes encoding MurA and MurB that are involved in the formation of UDP-N-acetylmuramate were down-regulated, with a fold changes of 1.8 for both. The *murC*, *D*, *E*, and *F* genes encoding cytoplasmic enzymes responsible for the sequential adjunction of amino-acids to UDP-N-acetylmuramate, leading to the formation of UDP-MurNAc-pentapeptide, were also down-regulated (with fold changes of 2.3, 1.6, 1.2, and 2, respectively) as well as the gene *ddlA* reponsible for the formation of the D-alanyl-D-alanine dipeptide is down regulated (fold changes of 2,9)*.* Two proteins MraY and MurG ensure the transfer of the phospho--MurNAc-pentapeptide moity of the UDP-MurNAc-pentapeptide to the membrane acceptor and the addition of GlcNAc, leading to the formation of GlcNAc-MurNAc-pentapeptide on the lipid carrier. The *mraY* gene encoding UDP- MurNAc-pentapeptide phosphotransferase was repressed with a fold change of 3.4, whereas expression of the *murG* gene remained stable. Finally, the enzymes responsible of the late steps of the biosynthesis of the PG were also down regulated: *fstI* (fold change of 1.9) and *mrc/ponA* (fold change of 1.5). Taken together, our results showed a down-regulation of peptidoglycan synthesis, which agreed with the growth arrest and entry into dormancy observed for CIRM-BIA138.

The CIRM-BIA138 strain produces a surface β-D-glucan polysaccharide [18]. A single *gtfF* gene is responsible for the synthesis of this polysaccharide*.* The *gtfF* gene is strongly induced in stationary growth phase compared to the exponential phase, with a fold change of 6.1, which might lead to an overproduction of surface β-D-glucan polysaccharide. The induction was confirmed with a fold change of 3.3 at 3d but not at 9d post-inoculation by RT-qPCR results. Such EPS production might protect the bacteria against the unfavourable conditions encountered in stationary phase and therefore prepare them for long-term survival, as has previously been shown in other food species such as *Oenococcus oeni* [18].

The cell wall of *P. freudenreichii* is also coated with proteins that are anchored via an SLH domain. SlpE, SlpF and SlpG are three of these proteins. Interestingly, during this study, the *slpE* gene was found to be strongly up-regulated (fold change of 12.9); whereas *slpG* and *slpF* were down-regulated (fold change of 2.8 and 2.2 respectively). According to RT-qPCR results, the induction of *slpE* is transient since it was confirmed at 3d with a fold change of 5.5 but not at 9d post-inoculation. As of yet, the physiological role of these proteins has not been elucidated, but these results suggest differing roles in long-term survival for SlpE and the other two proteins.

The bacterial membrane mostly comprises a protein-embedded phospholipid bilayer. In CIRM-BIA138, the fatty acid biosynthesis pathway was strongly repressed in stationary phase compare to exponential phase. The *fabF* and *fabH* genes encoding the enzyme charging the acetyl residue from acetyl-CoA to acyl-carrier protein (Acp) were repressed, with respective fold changes of 6.3 and 6.9. RT-qPCR results confirmed repression of *fabF* with a fold-change of 8.2 at 3d and 6.6 at 9d post-inoculation. The *acp* gene was repressed, with a fold change of 5.9. In the same way, the *fabD* genes encoding the enzyme charging the malonyl residue from malonoyl-CoA to Acp was repressed, with a 3.7-fold change. Genes such as *fabG* or *inhA,* which are responsible for the further elongation of fatty acids, were repressed by 1.9 and 1.7, respectively. Bacteria can produce fatty acids anaerobically. Since most fatty acids in bacterial cells are used for membrane phospholipid synthesis, growth arrest at the entry into stationary phase at 3d compared to 1d post-inoculation (see CFU count, Fig. 1) might limit the need for fatty acids, and lead to the down-regulation we observed here.

### Diversification of nutrients

At 3d post-inoculation, glucose, lactose, and lactate were exhausted. *P. freudenreichii* therefore needed to recruit other catabolic pathways to produce NADH and NADPH reducing equivalents, ATP and precursor metabolites required for the survival. Inositol and arabinose pathways were induced. Gene-set enrichment analysis revealed that “Pentose and glucuronate interconversions” were the most markedly induced KEGG pathways (ko00040; fold change of 1.7, pval 0.003). In this large pathway, the degradation of arabinose appeared to be the only one to be induced.

### Arabinose

Most *P. freudenreichii* strains can degrade L-arabinose, and the degradation pathway has previously been described [15]. L-arabinose enters the cell via a xylose/ribose/arabinose/galactoside ABC transporter encoded by *rbsBA* that is not differentially expressed. L-arabinose is sequentially converted to L-ribulose, L- ribulose 5-phosphate, and D-xylulose 5-phosphate by the action of the L-arabinose isomerase encoded by *araA*, L-ribulokinase encoded by *araB* and ribulose-5- phosphate 4-epimerase encoded by *araD* and *araD1*, which in our study were induced 2.2, 2.2, 1.1 and 2.9 fold, respectively. However, biochemical quantification failed to detect arabinose which is probably present at a concentration lower than the detection limit of 0.5 mM (like glucose, lactose, fructose, mannose) in the medium.

### Inositol

**Fig. 3:**
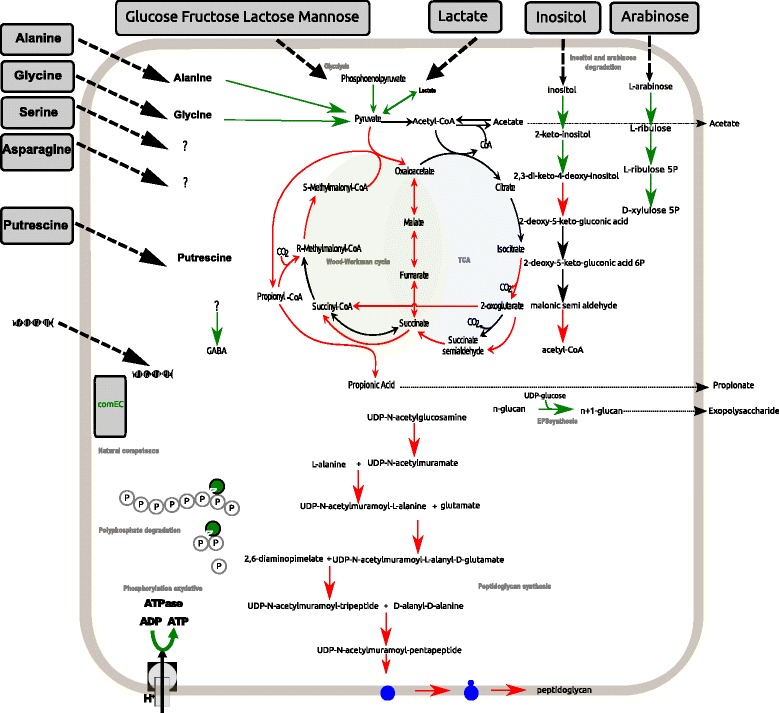
Schematic view of the metabolism at the entry into stationary phase compared to exponential phase. Induced/repressed/‘non differentially expressed’ genes are symbolized with green/red/black arrows respectively. (*P* value ≤0.005)

Inositol is a six-fold cyclohexane alcohol. Inositol is found in many foods (particularly in fruits) and is probably present in the yeast extract contained in YEL culture medium. Inositol is transported into the cell by a transporter encoded by *iolT1*, *iolT2* and *iolT3*, which were found to be induced with respective fold changes of 11, 3.2 and 1.7, at the entry into stationary phase compared to exponential phase (Additional file 2: Table S2). The induction of *iolT1* was induced 2.4 at 3d and 58.3 at 9d according to RT-qPCR results. In the cell, inositol is transformed into 2-keto-inositol by inositol dehydrogenase that is encoded by the *iol, iolG2* genes, induced with fold changes of 5.5 and 2.1, respectively. 2-keto-inositol is then transformed into 2,3-di-keto-4-deoxy inositol by 2-keto inositol dehydratase encoded by *iolE3*, which was found to be induced by a 2.2 fold change. 2,3-di-keto-4-deoxy inositol is then transformed into 2-deoxy-5-keto gluconic acid by the product of the *iolB* gene repressed with a fold change of 2.1. The *iolC* and *iolD* genes, whose products convert 2-deoxy-5-keto gluconic acid into malonic semialdehyde, were not differentially expressed. The induction of inositol degradation was not detected by GAGE, probably because the steps in this pathways are not fully described in the KEGG orthology map for *P. freudenreichii* species. The inositol degradation pathway had previously been annotated manually in all sequenced strains able to degrade inositol [15], and this revealed a high number of paralogous genes at different steps in the pathway (Fig. 3), suggesting that the pathway is essential for this species. During our study, we were able for the first time to demonstrate the expression and induction of the inositol degradation pathway in *P. freudenreichii*. Unfortunately, inositol was undetected in the culture medium (data not shown).

### Use of asparagine

**Fig. 4:**
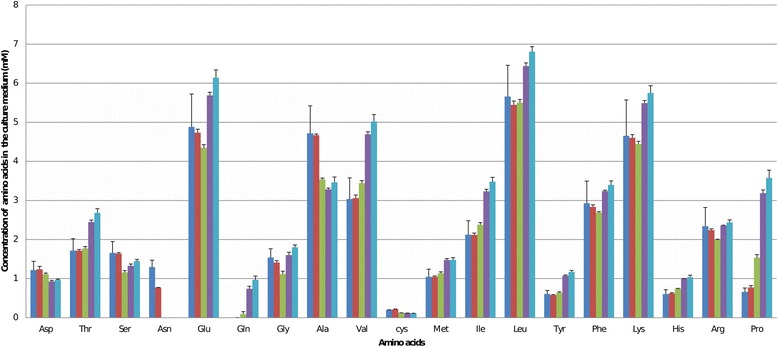
Concentrations of free amino acids in the medium (mM) during growth and stationary phases. In dark blue at the time of inoculation, in red at 1d post-inoculation, in green at 3d post-inoculation, in purple at 9d post-inoculation, in light blue at 11d post-inoculation. Significant consumptions discussed in the text (serine, asparagine, glycine, alanine) correspond to the difference in concentration at 1d and 3d post-inoculation (*t*-test, *P* value adjusted <0.05)

Biochemical analysis of the culture medium showed that asparagine was totally consumed between 1d and 3d post-inoculation (1.3 mM consumption, Fig. 4). The KEGG and Metacyc pathways report that in bacteria, asparagine is degraded into aspartate that is further transformed into fumarate or succinate. Despite asparagine exhaustion in the CIRM-BIA138 supernatant, the transcripts of *asmA* and *asmB* enabling the transformation of asparagine into aspartate were both repressed at 3d post-inoculation compared to 1d post-inoculation, with fold changes of 2.1 and 1.5, respectively. In *P. freudenreichii*, the fate of asparagine between 1d and 3d post-inoculation remains unknown [19]. The use of asparagine as an energy supply has previously been suggested [14]. Asparagine is described as being co-metabolized along with aspartate and lactate in type strain *P. freudenreichii* CIRM-BIA1 [20]. However, the quantification of free aspartate did not support this idea in the *P. freudenreichii* CIRM-BIA138 strain. The concentration of free aspartate in the medium was not significantly different between 1d and 3d post-inoculation (*t*-test, *P* value >0.05). The majority (i.e., 70% of the 100 *P. freudenreichii* isolates previously tested) displayed very low levels of aspartate activity [21]. In CIRM-BIA138, the lack of aspartate consumption could be explained by the lack of the *dcuA* gene enabling the transport of aspartate into the cell.

### Putrescine

Putrescine is a deleterious biogenic amine that is often present in mature cheeses. In general, Swiss-type cheeses containing the *P. freudenreichii* species do not exhibit high levels of putrescine [22]. Whether this is due to the degradation of putrescine by *P. freudenreichii* remains to be demonstrated. The degradation pathway for putrescine has not yet been described in *P. freudenreichii*. During the present study, the PFCIRM138_100015 gene, putatively encoding a putrescine importer, was induced with a fold change of 3.7 at entry into stationary phase. In *E. coli,* putrescine degradation is supported by the *gadA* and *gadB* gene products and involves steps that are shared with those of glutamate. The glutamate decarboxylase encoded by gadB was 5-fold more induced at 3d compared to 1d post-inoculation. RT-qPCR results confirmed a strong and progressive induction of *gadB* with a fold change of 3 at 3d and of 114 at 9d post-inoculation. The glutamate concentration did not differ statistically at 3d and 1d post-inoculation (*t*-test, *P* value >0.05), suggesting that degraded glutamate arose from the prior degradation of putrescine. This induction was also consistent with that observed in *E. coli* for the *gad*A and *gad*B genes during entry into stationary phase. It has been shown that glutamate decarboxylase activity increases the survival of *Listeria monocytogenes,* because intracellular glutamate is stored and converted into γ-aminobutyric acid (GABA), and extracellular glutamate is coupled to an antiporter in the *gad* system [23, 24]. The CIRM-BIA138 genome contains two genes encoding a GABA transferase: PFCIRM138_10030 (*gabT*), which was repressed with a fold change of 6.6, and PFCIRM138_04365, which was induced with a fold change of 10.6. However, neither GABA nor putrescine were detected in the medium despite a quantification limit of 25 μM (data not shown). The induction of putrescine importer (PFCIRM138_100015) and *gadB* at d3 and d9 is not sufficient to know whether these both genes were involved in the metabolism of stationary phase and whether glutamate metabolism could be linked to an hypothetical putrescine degradation by *P. freudenreichii.*

### Use of glycine

The dosage of free glycine in the medium revealed a 0.42 mM consumption between 1d and 3d post-inoculation (Fig. 4). The *dadA2* gene encoding glycine oxidase, which catalyses glycine oxidative deamination was induced at 3d compared to 1d post-inoculation, with a fold change of 13 according to RNAseq analysis but the induction was not statistically significant according to RT-qPCR result. This gene is the only one revealing a discrepency between RNAseq data and RT-qPCR analysis. However we can conclude to a maintain of *dadA2* expression at 3d and even at 9d post-inoculation. During osmotic stress induced by a high NaCl concentration in a medium containing glycine betaine (also named trimethylglycine), it was found that *Propionibacterium acidipropionici* accumulates glycine betaine in order to balance osmolarity and enable adaptation [24]. Other studies have also shown that in *P. freudenreichii*, glycine and proline exert protective effects against high osmolarity throughout bacterial growth [19]. Internalization of glycine could be a way to cope with osmotic conditions encountered at the entry of stationnary phase.

### Use of alanine

Alanine was the free amino acid that was most consumed (1.17 mM between 1d and 3d post-inoculation). This was in line with induction of the *ald* gene encoding an alanine dehydrogenase (2.7 fold change), which deaminates L-alanine into pyruvate. The induction of *ald* throughout growth has already been observed in stationary phase induced by low temperature in the absence of nutrient shortage [25]. We showed here that such *ald* induction also occurred in a context of nutrient shortage.

### The role of proline

Biochemical analysis between 1d post-inoculation and 3d post-inoculation an almost two-fold increase in proline was measured in the surpernatant (0.87 mM released) (Fig. 4). As no gene for proline synthesis was induced at 3d post-inoculation, this suggests that proline was released due to the degradation of proteins and peptides in the medium. This was consistent with the action of a proline iminopeptidase (Pip) which cleaves amino-terminal proline residues from peptides and has previously been described in cheese [26].

### Chaperone and detox

Protein chaperones protect other proteins against aggregation and denaturation. In *P. freudenreichii*, chaperones are strongly expressed in the case of acidic or bile salt stresses [27] or under cold conditions [25]. At 3d post-inoculation, the three sequences *hsp20 1*, *hsp20 2* and *hsp20 3* encoding heat shock proteins were induced with fold changes of 6.7, 6.9 and 2.5, respectively. The *copZ* gene encoding a copper chaperone was induced with a fold change of 2.5. The *cspB* gene encoding a cold shock protein was induced with a 2.7 fold change. The number of chaperones induced seems low when compared to the large number of chaperone-encoding genes described in the literature for *P. freudenreichii*. The limited induction of chaperone genes might reflect the efficient adaptation of *P. freudenreichii* to nutritional shortage. Alternatively, and unlike osmotic or heat-shock stress, nutritional shortage may not induce protein misfolding or denaturation and thus not cause the expression of many chaperone genes.

### Competence

In several species, natural competence enables the bacteria to cope with stresses or nutritional shortage. There are three prevailing hypotheses regarding the benefits of DNA uptake and recombination in bacteria (for a review, see [28, 29]): (i) DNA is used as a nutrient source; (ii) DNA is used to improve the efficiency of natural selection (by acquiring new genetic information); and (iii) DNA is used as a template to repair damaged chromosomal DNA. In the case of *P. freudenreichii* CIRM-BIA 138, a gene encoding DNA transfer protein (PFCIRM138_00630) was induced with a fold change of 14.7, and *dprA* encoding a DNA processing/uptake protein was induced with a fold change of 27.3 at 3d post inoculation. Two contiguous genes encoding mobilization protein (PFCIRM138_07015, PFCIRM138_07020) were induced with fold changes of 49.4 and 10.8 respectively, one gene (PFCIRM138_07570) encoding a conjugative relaxase was induced with a 24-fold change and lastly the comEC gene involved in competence was induced with a fold change of 17.4 at 3 d post-inoculation. PFCIRM138_07015 induction was confirmed by RT-qPCR result with a fold change of 23.6 at 3d and 26.7 at 9d post-inoculation. The non coding RNA named ‘Alil pseudoknot ‘was induced with a fold change of 3 at the entry into stationary phase (Additional file 1: Table S1). It is known to stimulate the expression of transposase, an enzyme required for transposition. *P. freudenreichii* is known to have evolved more by recombination than by mutations [30] and possesses a high copy number of insertion sequences and integrase genes in its genome [7]. Here, several genes involved in natural competence were found to be induced under the conditions applied. However, neither natural competence nor conjugation has been described in this species to date. Cultures of CIRM-BIA138 in presence of high concentration of insertional plasmid (data not shown) failed to illustrate natural competence.

### Utilization of pyrophosphate

*P. freudenreichii* can accumulate inorganic polyphosphate (polyP) as an energy reserve whereas most bacteria utilize ATP [7]. The ability to use polyphosphate as an energy reserve has been shown to be specific to bacteria adapted to difficult environments. The CIRM-BIA138 genome possesses 20 genes encoding enzymes which use polyphosphate or pyrophosphate. At the start of the stationary phase during our study (at 3d post-inoculation), three phosphorylases were induced when compared to exponential growth phase at 1d post-inoculation: (i) *ppx5,* encoding an exopolyphosphatase with a 19.8 fold change, (ii) *ppa* encoding an inorganic pyrophosphatase with a 1.6 fold change, and (iii) PFCIRM138_07685 encoding a NUDIX hydrolase with a 3 fold change. The *phoH* gene, encoding a phosphate-starvation inducible protein, was induced with a 2.5 fold change. In *Corynebacterium glutamicum*, another Actinobacterium that is phylogenetically close to *P. freudenreichii*, previous microarray results showed an induction of the *phoH* gene with a fold change ranging from 1.1 to 6.8 under Pi-limiting conditions compared to non-limiting conditions [31]. In many species, strains with a mutation in the gene involved in polyphosphate (*ppk*) synthesis are unable to survive during stationary phase [32]. Taken together, these results suggested a possible limitation of the availability of soluble phosphate causing *P. freudenreichii* to use polyphosphates stored in its cytoplasm during stationary phase to cover its phosphate requirements. Since the phosphate starvation response is critical for the persistence of *Mycobacterium tuberculosis* (another Actinobacterium) in the lung [33], the role of phosphate starvation in the entry of *P. freudenreichii* into dormancy needs to be further explored, e.g., by quantifying extracellular phosphate and intracellular polyphosphate and by directed mutagenesis on key enzymes in the polyphosphate synthesis pathway.

## Conclusions

At entry into stationary phase, *P. freudenreichii* adapts its metabolism to nutritional shortage and slows down its metabolism. Genes involved in oxidative phosphorylation and fermentation (via the WoodWerkman cycle) are repressed, in line with the lack of lactate in the medium, enabling the entry into dormancy. By contrast, *P. freudenreichii* diversifies its source of nutrients and appears to utilize amino acids which differ from those used during the exponential phase according to RNAseq analysis, RT-qPCR and amino acids quantification. To meet its energy needs *P. freudenreichii* probably utilizes polyphosphate, because several phosphatases were found to be induced during this study. These results therefore provide an analysis of *P. freudenreichii* adaptation during entry into stationary phase by means of comprehensive gene expression analysis using RNA-Seq combined with targeted biochemical quantifications. They shed light on important molecular mechanisms that might be involved in the long-term survival of *P. freudenreichii*, and open avenues for further investigation on its survival strategies.

## Methods

### Bacterial strains

*P. freudenreichii* CIRM-BIA138 (alias ITG P9) strain was used during this work. The strain was supplied by the International Centre for Microbial Resources-Bacteria of Food Interest (Centre International de Ressources Microbiennes-Bactéries d’Intérêt Alimentaire; INRA, Rennes).

### Conditions for bacterial growth

The strain was cultured in YEL medium (pH = 7.0) [34] with no agitation at 30 °C for 11 days. The strain was grown in a different assay tube for each manipulation, thus preventing any interference of oxygen in the environment and maintaining the strain under microaerophilic conditions. At T0, the medium was inoculated at10^7^ CFU/mL and the inoculated culture was then split into 5 tubes of 10 ml at T0.

The growth kinetics were followed using Optical Density measurements with a Model DU 640 spectrophotometer (Beckman Coulter, Fullerton, California, USA) at 650 nm (OD_650_), and the CFU (CFU/mL) were counted using the micromethod described previously [35]. *P. freudenreichii* enumerations were carried out on YEL agar at 30 °C under microaerophilic conditions, until the visualization of colonies (6 days). Two technical replicates and two biological replicates were performed for growth curves analysis, and three biological replicates were performed for RNA-seq and biochemical analysis. Aliquots of the culture were sampled for RNA extraction, pH measurements and biochemical analysis (see above) on the day of inoculation and at 1d, 3d and 9d post-inoculation.

### RNA extraction and quality control

1 ml of each culture was mixed with 2 volumes of RnaProtect (Qiagen, Hilden, Germany), left for 5 min at room temperature and then centrifuged (8,000 g, 10 min at room temperature). The supernatant was removed and the pellet stored at -80 °C until total RNA was extracted. The pellets were thawed on ice, suspended in 200 μL lysis buffer (50 mM Tris–HCl, 1 mM EDTA; pH 8.0) containing 20 mg/mL lysozyme (MP Biomedicals, Illkirch, France) and 50 U/mL mutanolysin (Sigma, Saint Quentin Fallavier, France), and incubated for 15 min at 24 °C. The suspensions were then transferred to two millilitre tubes containing 50 mg zirconium beads (diameter: 0.1 mm; BioSpec Products, Bartlesville, OK) and 100 μL SDS (10%). The tubes were shaken twice for 90 s at 30 Hz with a bead beater (MM301; Retsch, Haan, Germany), being chilled on ice for 2 min. between the shaking steps. RNA extraction was then performed using an RNeasy minikit (Qiagen) and the Qiacube extraction robot (Qiagen), according to the manufacturer’s instructions. RNA were suspended in 50 μL RNase-free water and treated with DNase (DNA-free; Ambion, Cambridgeshire, United Kingdom) according to the supplier’s instructions, and then stored at -80 °C until use. RNA was then quantified and the contamination of RNA by proteins was assessed spectrophotometrically using a NanoDrop ND-1000 spectrophotometer (NanoDrop Technologies, Inc., Rockland, DE, USA). RNA quality was evaluated using an Agilent 2100 Bioanalyzer (Agilent Technologies, Santa Clara, CA, USA). All the RNA samples from spent medium had a RIN value higher than 7.5, indicative of good rRNA integrity. RNA was then depleted using RiboZero Magnetic kit for Gram positive bacteria (Epicentre, Madison,WI, USA) according to the manufacturer’s instructions. Depleted RNA quality was evaluated using an Agilent 2100 Bioanalyzer (Agilent Technologies, Santa Clara, CA, USA) and nanoarrays for prokaryotes.

### Sequencing of mRNA

The cDNA libraries were prepared from the depleted RNA for each of the three repetitions of the exponential phase (1d post-inoculation), stationary phase (3d post-inoculation) and long-term survival (9d post-inoculation) and then prepared for sequencing using the Illumina TruSeq Stranded mRNA LT Sample Preparation kit. The nine oriented RNA-seq libraries were prepared using the TruSeq Stranded mRNA Sample Preparation kit, according to the manufacturer’s instructions. Qualitative and quantitative library validations were performed on a DNA 1000 Agilent chip as well as using quantitative PCR with SYBR Green (Applied Biosystem 7500). The libraries were sequenced on an Illumina HiSeq 2000 as 50 bp reads using the Sequence By Synthesis technique at the Montpellier GenomiX facility. Image analyses and base calling were performed using the Illumina HiSeq Control Software and Real-Time Analysis component. Demultiplexing was performed using Illumina’s sequencing analysis software (CASAVA 1.8.2). Data quality was assessed using FastQC from the Babraham Institute and the Illumina software SAV (Sequencing Analysis Viewer). The concentration in the samples corresponding to long-term survival (9d post- inoculation) were too low for sequencing and revealed contamination with genomic DNA, so they were not further analysed.

### RNA-seq analysis

Sequences were mapped with Bowtie [36]. To detect non-coding RNA, mapped sequence files where pooled by phase (exponential or stationary). Then, each file was submitted to Detr’Prok [37] to detect positions of small RNA, antisens RNA and 5′ Untranslated regions. Bedtools [38] is used to extract corresponding sequences. Sequences where submitted to Rfam [39] to eliminate non characterized sequences. Finaly, non coding RNA validated by Rfam and CDS were counted with htseq-count [40] using the ABIMS Roscoff platform. A list of differentially expressed genes was generated using an R software package: SARtools [41], embedded Deseq2 [42] and EdgeR [43] (modified *t*-test adjusted, Pvalues < 0.05). A comparison of the results obtained with either Deseq2 or EdgeR produced a more exhaustive list using EdgeR. Most of the differentially expressed genes from Deseq2 were present in the EdgeR results. For this reason, only the results obtained using EdgeR are discussed below.

The protein sequences of the strain were subjected to a search against the Kyoto Encyclopedia of Genes and Genomes Pathway database (KEGG) using GhostKOALA (http://www.kegg.jp/ghostkoala/) to retrieve KEGG orthology (KO) identifiers. 1143 out of 2304 genes were annotated automatically. Differentially expression data were associated with these KO identifiers and underwent gene set enrichment analysis on KEGG orthology pathways using the GAGE package [44] of R software to highlight pathways that were significantly differentially expressed (pval <0.1).

### RT-qPCR validation

*cstA*, *eno1* and *sdaA* were used as housekeeping genes. Relative quantification relates the PCR signal of the target transcript at 3d and 9d post-inoculation compared to that of the inoculation time used as control. Before RT-qPCR, a supplementary DNAse treatment was applied to total RNA from 0, 3d and 9d post-inoculation samples. cDNA were obtained from a retrotranscription step using iScript cDNA synthesis kit (Bio-Rad, Marne la Coquette, France). cDNAs were amplified as follows: each PCR mixture included 5 μl of 1/50 diluted cDNA, 3 μl of 200 nM of primers, 8 μl of IQ Sybr Green supermix (Bio-Rad, Marne la Coquette, France). Amplifications were carried out on a CFX96 Real Time System (Bio-Rad) for 5 min at 95 °C and 40 cycles of 2 steps consisting of 15 seconds at 95 °C and 30 seconds at 60 °C. The relative quantification of the mRNA levels of the target genes was determined using CFX Manager (Bio-Rad, Marne la Coquette, France). The amount of target was normalized to *cstA*, *eno1* and *sdaA* genes because they were revealed as stably expressed according to the software: Delta C_q_ = C_q_ (target gene) – C_q_ (housekeeping gene), where C_q_ represents the cycle number required to reach a defined threshold target abundance.

### Metabolome analysis

The samples used for this study had previously been frozen at -20 °C. These analyses were performed on the following samples: (i) YEL culture medium, (ii) exponential (1d post-inoculation), (iii) stationary (3d post-inoculation), (iv) late stationary (9d post-inoculation) and (v) long-term survival (11d post-inoculation), with three biological replicates of each sample. The YEL culture medium was used as a control.

- **Amino acid assays proteins and peptides**. in the samples were precipitated by adding solid sulphosalicylic acid to 5% (w/vol), holding at 4 °C for 1 h, and centrifuged (5,000 g, 15 min). The supernatant was filtered through a 0.45 μm pore size filter and diluted with a 0.2 mol/L lithium citrate buffer (pH 2.2) before injection. Amino acid analyses were then carried out using cation exchange chromatography on a Biochrom 30 AA analyser (Biochrom Ltd, Cambridge, UK) according to the method described by Moore et al. (1958) with lithium citrate buffers as eluents and the ninhydrin post-column reaction system. Data were recovered using the control software Biosys and chromatographic data were processed with EZChrom Elite.
- **Quantification of lactate, pyruvate, acetate, citrate and propionate**. lactic, propionic, acetic, succinic and pyruvic acids were quantified in culture supernatants using an HPLC Aminex A-6 ion exchange column (Bio-Rad, Hercules, CA, USA) at 60 °C with 0.005 M H2SO4 at an eluent flow rate of 0.4 ml min-1. Acids were detected by UV (210 nm) and/or refractometry (RI2031 plus, Jasco).
- **Quantification of arabinose and inositol**. Concentrations were measured in culture supernatants by high performance liquid chromatography (Agilent Technologies 1200 Series, Waldbronn, Germany) using a HPX87H^+^ Biorad column and the following conditions: a temperature of 48 °C, eluent H2SO4 (5 mM) at a flow rate of 0.5 mL/min, and dual detection (refractometer and UV).
- **Quantification of glucose, mannose, fructose and lactose**. The supernatant of culture medium was deproteinized using Vivaspin 10 kDa filters. Glucose, mannose, fructose, and lactose were separated on a CarboPacMA1 (4 × 250 mm) analytical column (preceded by a corresponding guard column 50 × 4 mm) with 16 mM NaOH as the eluent and a flow rate of 1 mL/min using an ICS-3000 chromatography/detector module (Dionex) and a range of 1 μC. A solution containing each of the four sugars (glucose, mannose, fructose and lactose) (Sigma-Aldrich) at 2, 5,10, 20 and 40 mg/L (linearity range) was used as a standard. Detection and quantification were performed using amperometry and expressed in μC.

## Additional files

Additional file 1: Table S1. List of repressed genes in stationary phase (3d post-inoculation) compared to exponential phase (1d post-inoculation) (*P* value adjusted ≤0.05). (XLSX 96 kb)
Additional file 2: Table S2. List of genes induced in the stationary phase (3d post-inoculation) compared to the exponential phase (1d post-inoculation) (*P* value adjusted ≤0.05). (XLSX 98 kb)
Additional file 3: Table S3. RT-qPCR results for validation of RNAseq results. (XLSX 9 kb)

## Abbreviations

CFU: Colony forming unit
EPS: Exopolysaccharide
GlcNAc: N-acetylglucosamine
LTS: Long-term survival
MurNAc: N-acetylmuramic acid
PG: Peptidoglycan
RNAseq: Ribonucleic acid sequencing
RT-qPCR: Retrotranscription quantitative polymerase chain reaction
UTR: Untranslated region
YEL: Yeast extract lactate
